# Characteristics and Dynamics of Full Arch Distalization Using Transpalatal Arches with Midpalatal and Interradicular Miniscrews as Temporary Anchorage Devices: A Preliminary Finite Element Analysis

**DOI:** 10.1155/2020/6648526

**Published:** 2020-12-18

**Authors:** Mashallah Khanehmasjedi, Sepideh Bagheri, Vahid Rakhshan, Mojtaba Hasani

**Affiliations:** ^1^Dept of Orthodontics, Dental School, Ahvaz Jundishapur University of Medical Sciences, Ahvaz, Iran; ^2^Dept of Anatomy, Dental School, Azad University of Medical Sciences, Tehran, Iran; ^3^Mechanical Engineering Department, Iran University of Science and Technology, Tehran, Iran

## Abstract

**Introduction:**

Miniscrews have proved quite effective in fixed orthodontic treatment. They can be placed in areas like palatal interradicular zones or midpalatal suture. Despite the value of these methods and their ever-increasing use, their characteristics are not assessed before when implanted in palatal interradicular areas or in the midpalatal suture. We aimed to assess, for the first time, the dynamics of full arch distalization using such miniscrews.

**Methods:**

A 3D model of maxilla with all permanent dentition was created from a CT scan volume. Tissues were segmented and differentiated. Afterward, miniscrews and appliances were designed, and the whole model was registered within a finite element analysis software by assigning proper mechanical properties to tissues and orthodontic appliances. The full arches were distalized using transpalatal arches with miniscrews as anchorage devices (in two different models). The extents of stresses and patterns of movements of various elements (teeth, miniscrews, appliances, tissues) were estimated. *Results and Conclusions*. Comparing the two models, it is obvious that in both models, the stress distribution is the highest in the TPA arms and the head of the miniscrew where the spring is connected. In comparison with the displacement in the *X-axis*, the “mesial in” rotation is seen in the first molar of both models. But there is one exception and that is the “mesial out” rotation of the right second molar. In all measurements, the amount of movement in Model 2 (with palatal interradicular miniscrews) is more than that in Model 1 (with midpalatal miniscrew). In the *Y*-axis, more tipping is seen in Model 2, especially the anterior teeth (detorque) and the first molar, but in Model 1, bodily movement of the first molar is more evident. Along the *Z*-axis, the mesial intrusion of the first molar and the distal extrusion of this tooth can be seen in both models. Again, the displacement values are higher in the second model (with interradicular miniscrews). In comparison with micromotion and stress distribution of miniscrews, in Model 1, maximum stress and micromotion is observed at the head of the miniscrew where it is attached to the spring. Of course, this amount of micromotion increases over time. The same is true for Model 2, but with a lower micromotion. As for the amount of stress, the stress distribution in both miniscrews of both models is almost uniform and rather severe.

## 1. Introduction

Treatment options for both types of classes II and III malocclusions include growth modification, camouflage treatment, and orthognathic surgery. In complex cases of Class III malocclusion, orthognathic surgery is a priority, especially when the patient has a family history [1]. Surgical treatment in the preoperative phase requires decompensation [1]. Distalization is successful treatment and means a retraction of the entire maxillary arch, which can be used in the treatment of class II patients with increased overjet or anterior crowding, or in the orthodontic stage before orthognathic surgery in some Class III patients to increase the negative overjet to prepare them for orthognathic surgery [2–4]. En-masse distalization is one of the most successful treatment plans to correct the class II relationship [5–7].

In fixed orthodontic treatment, the presence of proper anchorage is one of the important factors in achieving the desired results and is a necessary requirement for the treatment of dental and skeletal malocclusions [8]. Anchorage is defined as resistance to unwanted movements of the tooth [8–10] and is needed for treating dental and skeletal malocclusions [11–13]. Achieving maximal or absolute anchorage is always a major challenge for the orthodontist, and loss of anchorage reduces the success of sagittal correction [14].

A desirable anchorage is an absolute anchorage: The use of structures other than teeth as anchorage allows therapeutic movements or growth modification to be performed without adverse effects [15]. There are currently several ways to provide absolute anchorage. The most basic of these is the use of titanium screws that are implanted into the bone after passing through the gingiva, and the other is the bone anchors that are usually placed in the bed of the zygomatic arch. Recently, the use of temporary anchorage devices (TAD) has become very common due to the delivery of absolute anchorage, fewer complications, and reducing dependence on patient cooperation, which make them proper for use in distalizing the whole arch in nonextraction treatment [16, 17]. The use of TADs has even reduced the need for extraction and surgical treatments [18]. Recently, miniscrew implants have been increasingly used in orthodontics due to the provision of absolute and skeletal anchorage for dental movements [19, 20], simple placement and removal without irreversible changes [21], immediate loading [22], low cost, short treatment time [23], minimal need for patient cooperation, and possibility of placing them in various anatomical sites due to their relatively small diameter (1.2 to 2 mm) [24, 25].

Miniscrews can be placed in some common places. One of the most successful places to implant a miniscrew is the buccal and palatal plates of the alveolar maxilla. However, the survival rate of implants placed in the alveolar ridge needs to be improved due to variations in bone quality and risk of root contact [26]. Interradicular screws need to be repositioned for successful distalization, while the maxillary buccal bone, especially in younger people, does not have the ideal quality for a miniscrew. However, the palatal bone is a safer area for the use of mini-implants, with a denser bone [27–29]. The midpalatal suture is a very suitable place with proper keratinized tissue, fully dense bone, and sufficient support to place a miniscrew. Besides, it does not have any dental root or significant vessels or nerves, making it a proper site for mini-implanting without the need for any surgical procedures. Currently, the midpalatal miniscrews can be used for retraction of the anterior maxillary teeth, intrusion, distalization, and protraction of the posterior teeth with high success, and they have made possible dental movements that were at best difficult with ordinary orthodontic mechanics [30, 31].

Despite the frequency of using these locations for orthodontic mini-implanting, the characteristics of these methods remain unassessed. Therefore, the purpose of this experimental laboratory study was to compare the stress distribution and displacement of maxillary teeth in three dimensions under the influence of total arch distalization with the help of miniscrews in two methods: (1) Midpalatal miniscrew (2 mm in diameter and 7 mm in length) with TPA on the first molars; (2) Palatal miniscrews placed between teeth 5 and 6 (diameter 1.8 mm and length 11 mm) with TPA on the first molars.

## 2. Materials and Methods

This in silico simulation was performed on a 3D model of the maxilla. The study protocol was approved by the research committee of the university in July 2020. The 3D model was created using a retrospectively taken CT scan, and therefore no human was exposed to X-ray.

In a finite element analysis, it is necessary to first prepare a 3D model of the maxilla, miniscrew, miniplate, and the maxillary arch, and using finite element software, the model components are superimposed on each other so that it can be considered as an integrated system. To do this, first, the geometric maxillary model was generated using CT scan data (NewTom VGi; Verona, Italy). Following the virtual modeling process, the images were transferred to Mimics 20 software (Materialize; Leuven, Belgium). In this software, 2D photos taken from the three main views of the frontal, sagittal, and transverse from the jaws are assembled on top of each other in a 3D environment to obtain a 3D view of the object. Due to the distance between each image and the next CT scan image, the assembled 3D model has noise. However, the shorter the distance between the images is (the higher the accuracy of the device), the less noise the 3D model will have. CT scan volumes were denoised in 3-Matic software (Materialize; Leuven, Belgium). Afterward, the 3D geometry of the maxilla was entered into SolidWorks software (version 2018, Dassault Systemes; Paris, France) to make appropriate geometric changes and assemble different parts in 3D. The 3D model was then transferred to the Ansys Workbench 2018 software (ANSYS Inc; USA), and the mechanical properties of all components that determine their mechanical behavior are applied to them.

After applying the properties of the components, their networking, which is one of the main parts of finite element analysis, was performed. To do this, our model was divided into smaller 3D parts called elements, which were formed by the juxtaposition of a number of nodes. The next step was to apply the boundary conditions, in which the fixed parts of the model were specified and forces were applied to the model. Afterward, calculations were performed. In the next step, displacement data, geometric data, and force data such as stress, strain, and displacement were extracted and displayed as data or tension contours.

### 2.1. Modeling of Maxilla and Separation of Cortical and Spongy Bones in Mimics and 3-Matic Software

**Figure 1:**
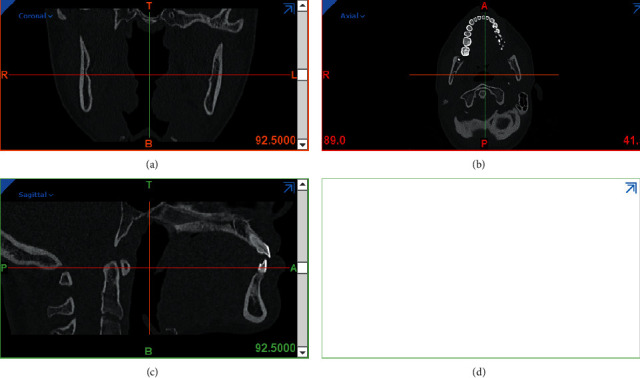
The CT scan used in this study.

First, bone, tooth, and TPA models were modeled in Materialize Mimics Innovation suite 21.0 and 3-Matic 13.0. CT scans of the jaw and face of a 30-year-old patient with a distance of 1 mm between the slices were entered into Mimics (Figure 1). In this project, 2 models of maxilla were designed. In Model 1, the midpalatal miniscrew (2 mm in diameter and 7 mm in length) was designed with TPA on teeth number 6, and in Model 2, the palatal interradicular miniscrew (1.8 mm in diameter and 11 mm in length) was designed with TPA on first molars. Then all parts were exported in STL format from these software programs.

**Figure 2:**
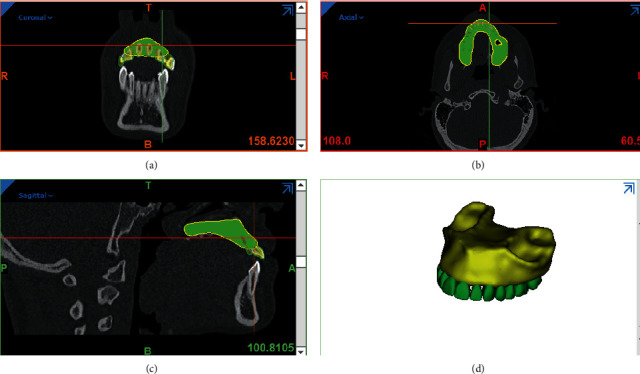
Segmentation of bones and teeth in the CT scan volume.

Using segmentation tools, masks for maxilla and PDL teeth and bones were created, and then a three-dimensional model of these components was created using the Calculate 3D command (Figure 2).

The orthodontic wire and bracket were modeled in 3-Matic software.

### 2.2. Design of Miniscrews

**Figure 3:**
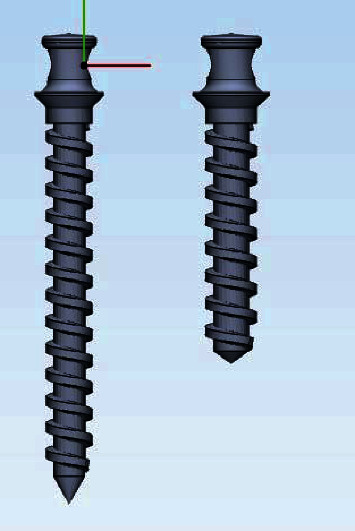
Miniscrews used in this study.

The SolidWorks software (version 2018, Dassault Systemes; Paris, France) was used to design the screws. The screw specifications were designed and applied based on what was given. With the help of Helix and Sweep commands and drawing the screw profile in SolidWorks software and defining the step and length of the miniscrews, these parts were created. The final model of the screws is given below (Figure 3).

### 2.3. Geometry Conversion in Geomagic Software

Parts exported in STL format from Mimics and 3-Matic software were converted to parts in STP format using Geomagic software (Designx 12 Geomagic, North Carolina, United States).

### 2.4. Analysis in ANSYS Software

**Figure 4:**
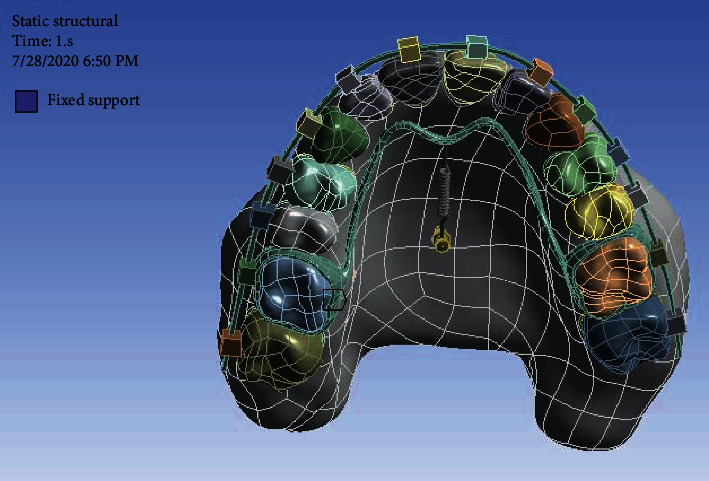
The reconstructed maxilla in ANSYS.

After converting all geometries to STP format, these geometries were entered into ANSYS software (Analysis systems 02/17, Canonsburg, Pennsylvania, United States) for analysis (Figure 4).

### 2.5. Boundary Conditions

**Figure 5:**
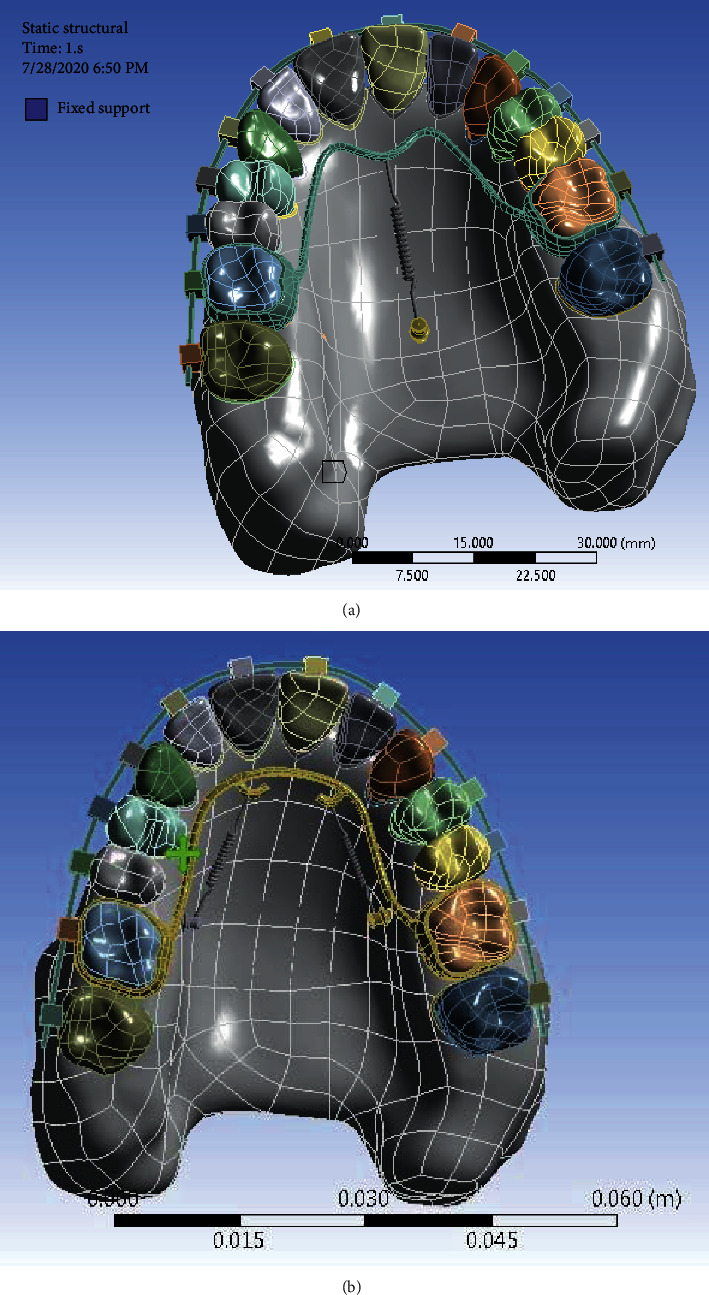
A force of 200 g was applied in both models.

A force of 200 g was applied to the spring that was attached to the TPA and the miniscrews, and the upper surface of the maxilla was fixed (Figure 5).

### 2.6. Mesh

**Figure 6:**
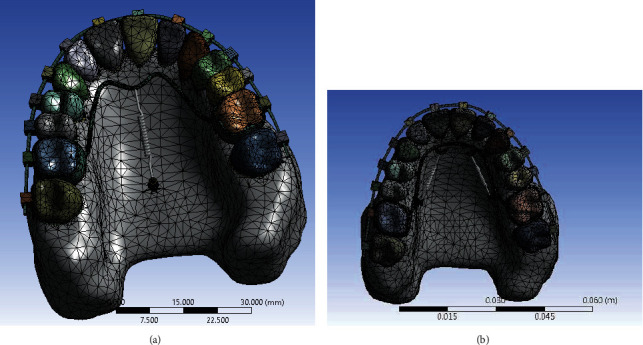
The mesh format of both models.

The total number of elements was 129237 tetrahedral elements and the number of nodes was 247282 (Figure 6).

### 2.7. Materials Specifications

The material properties of the different sections were defined according to previous articles [32, 33] (Table 1).

The ratio of lateral (transverse) to axial (longitudinal) strain is called the Poisson ratio, and the Young's modulus or modulus of elasticity is the ratio of the stress (force on an object per unit area) to the strain (change in the length of the body in any direction relative to the length of the body in the same direction) of linear solids are said to be below the yield strength (the amount of stress required to cause plastic deformation) [32, 33].

## 3. Results

### 3.1. Stress Distribution and Displacements

#### 3.1.1. Model 1

**Figure 7:**
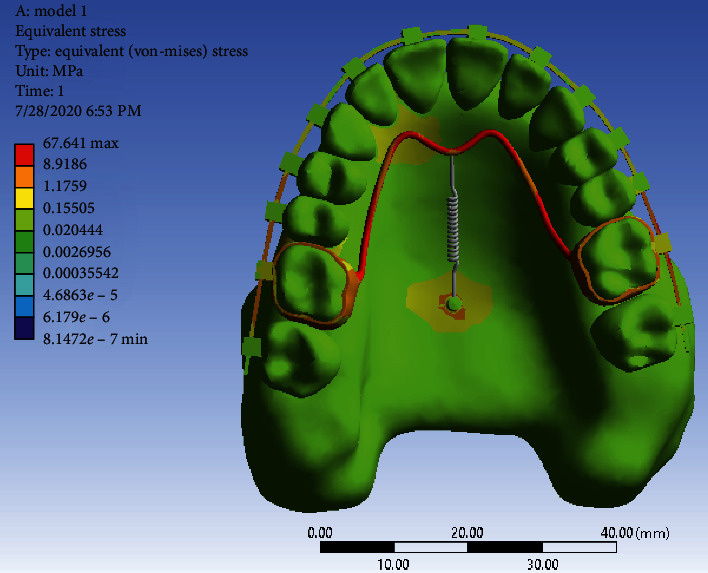
Stress distributions in Model 1.

Stress distribution: Stress changes in different parts of each jaw tooth were shown as a color diagram in the analysis. As shown in Figure 7, the maximum tension is seen in the TPA arms, followed by the molar band and the miniscrew head. There is also a lot of stress in the left palatal area of teeth 2 and 3. Other areas are green with minimal stress.

**Figure 8:**
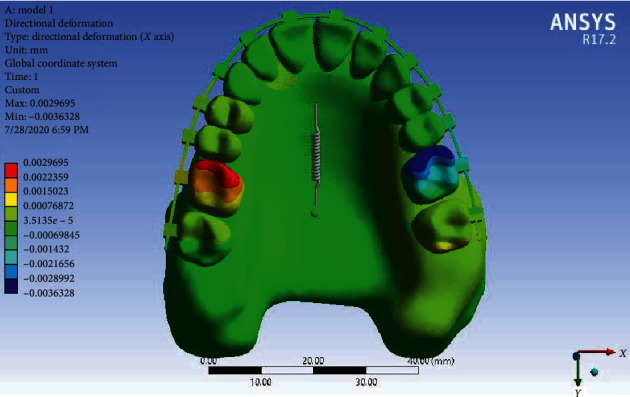
Displacement of Model 1 in the *X*-axis.

Displacement along the *X*-axis: According to Figure 8, in Model 1 in the *X*-axis, the displacement of the teeth is shown as rotation. In this model, at the same time as distalizing the maxillary arch along with the midpalatal miniscrew, first molar teeth are mesial in rotated; i.e., the mesial side of the tooth rotates inward. This mesial rotation is also seen in the second molars, but it is more visible in the first molars.

**Figure 9:**
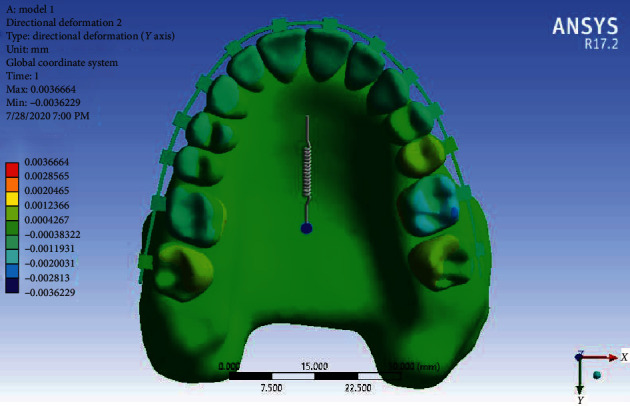
Displacement of Model 1 along the *Y*-axis.

Displacement in the *Y*-axis: Displacement in the *Y*-axis indicates the amount of tipping or body movement of the teeth. According to Figure 9, there is no obvious tipping, especially in incisors; and the teeth, especially the first molars, have moved in a bodily way. Of course, a small amount of tipping in one of the second premolars is visible.

**Figure 10:**
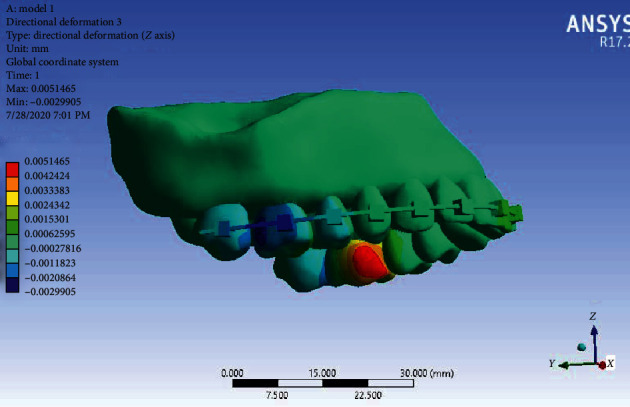
Displacement of Model 1 in the *Z*-axis.

Displacement along the *Z*-axis: Displacement in the *Z-axis* also indicates intrusion or extrusion of the teeth. With this explanation, in Figure 10, the mesial sides of the first molars on both sides are intruded and their distal sides are extruded. The greatest effect and displacement are on the second premolars and the first and second molars on both sides, but there is a significant amount of movement in the first molars.

#### 3.1.2. Model 2

**Figure 11:**
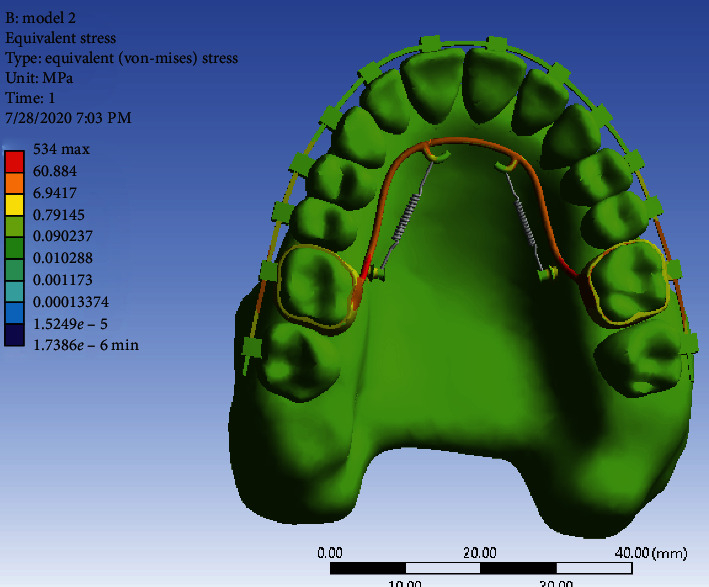
Stress distribution in Model 2.

Stress distribution: As can be seen in Figure 11, the highest amount of stress is seen in the TPA arms, especially in the vicinity of the miniscrew head and then in the first molar bands (similar to the first model). Other areas of the jaw are green with minimal stress.

**Figure 12:**
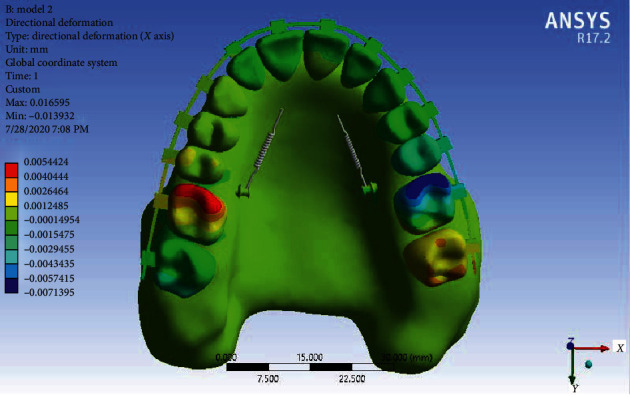
Displacement of Model 2 along the *X*-axis.

Displacement in the *X*-axis: Figure 12 shows the displacement of the teeth in the form of rotation in Model 2 in the *X*-axis. In this model, at the same time as distalizing the maxillary arch, the first molar teeth were “mesial in” rotated (the mesial side rotates inward). The notable subject is the “mesial out” rotation of the second molar tooth on the right (the mesial side rotates outward). Of course, the amount of rotation of this tooth is half of the first molar, but in the left second molar, the rotation is similar to the first molar and, of course, much less visible. In this model, slight rotations in the premolars are also seen.

**Figure 13:**
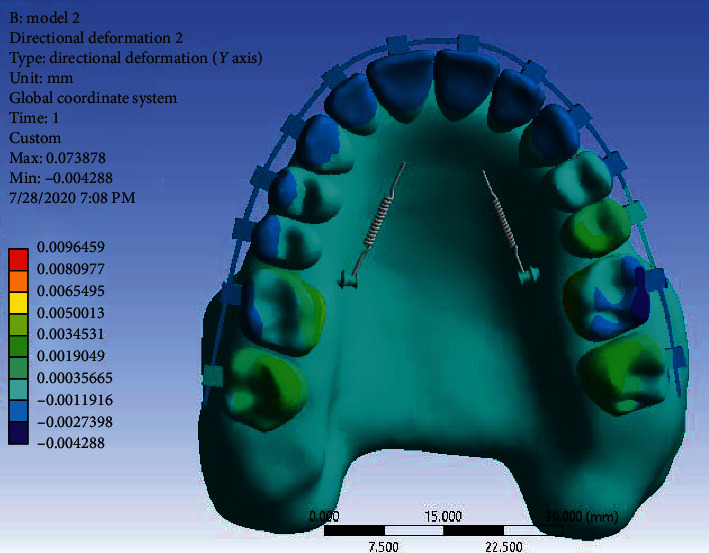
Displacement of Model 2 in the *Y*-axis.

Displacement along the *Y*-axis: Displacement in the *Y*-axis indicates the amount of tipping or body movement of the teeth. According to Figure 13, unlike the first model, there is clear tipping in the incisors, and the teeth in this model have more tipping and less bodily movement. The tipping of the incisors has caused them to be detorqued (moving the crown of the teeth to the palatal).

**Figure 14:**
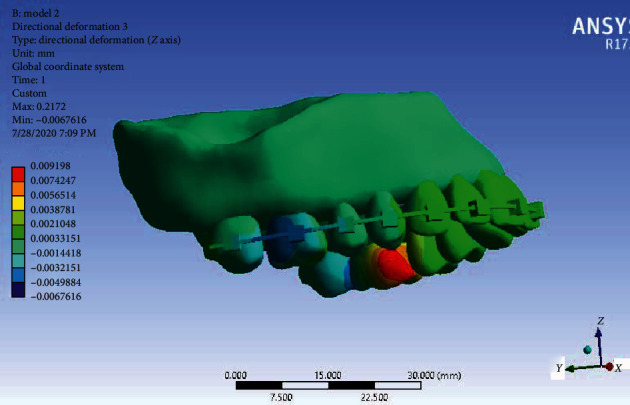
Displacement of Model 2 along the *Z*-axis.

Displacement in the *Z*-axis: The mesial sides of the first molars were intruded on both sides and their distal sides were extruded (Figure 14). The greatest effect and displacement were seen on the second premolars and the first and second molars on both sides, but the movement in the first molars was significant.

### 3.2. Micromotion and Stress Distribution of Miniscrew

#### 3.2.1. Model 1

**Figure 15:**
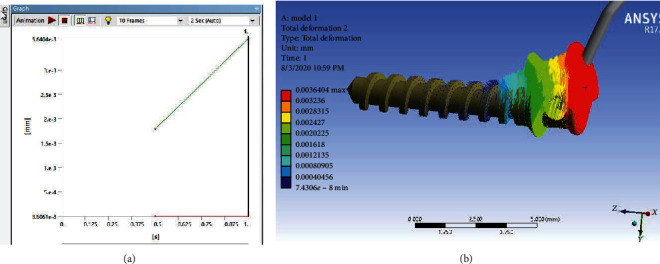
Micromotion of the miniscrew in Model 1 that increases over time.

**Figure 16:**
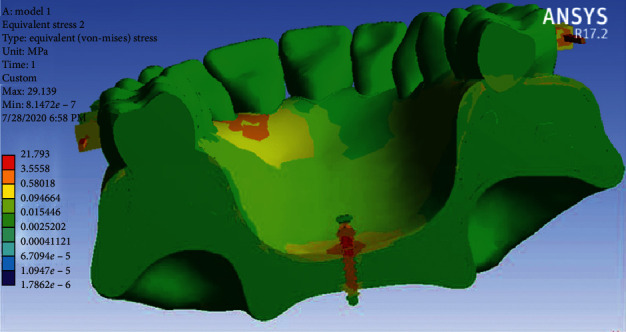
The stress distribution of miniscrew in Model 1.

According to Figures 15 and 16, maximum stress and micromotion were observed at the head of the miniscrew where it is attached to the spring. Of course, the diagram in Figure 15 shows that this amount of micromotion increases over time.

#### 3.2.2. Model 2

**Figure 17:**
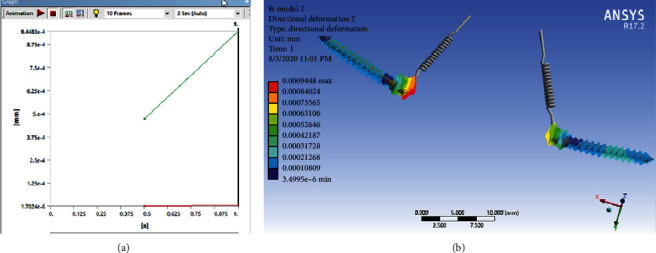
Micromotion of the miniscrew in Model 2 that increases over time.

**Figure 18:**
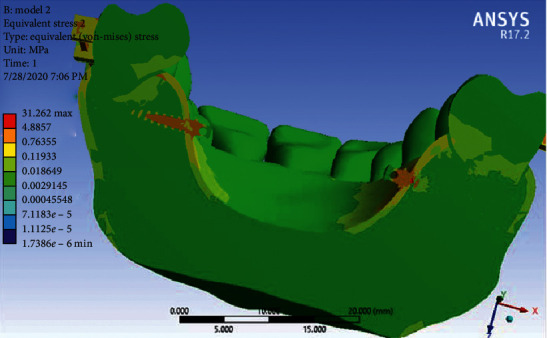
The stress distribution of miniscrew in Model 2.

According to Figures 17 and 18, in Model 2, the maximum micromotion was observed at the head of the miniscrew where it is connected to the spring. Of course, the diagram in Figure 17 shows that this amount of micromotion increases over time. But this amount of movement is less than that in Model 1. But in terms of stress, the stress distribution in both miniscrews is almost uniform and orange colored, which of course means that stress is seen in approximately 2/3 of the lengths of miniscrews.

### 3.3. General Displacement Contour

#### 3.3.1. Model 1

**Figure 19:**
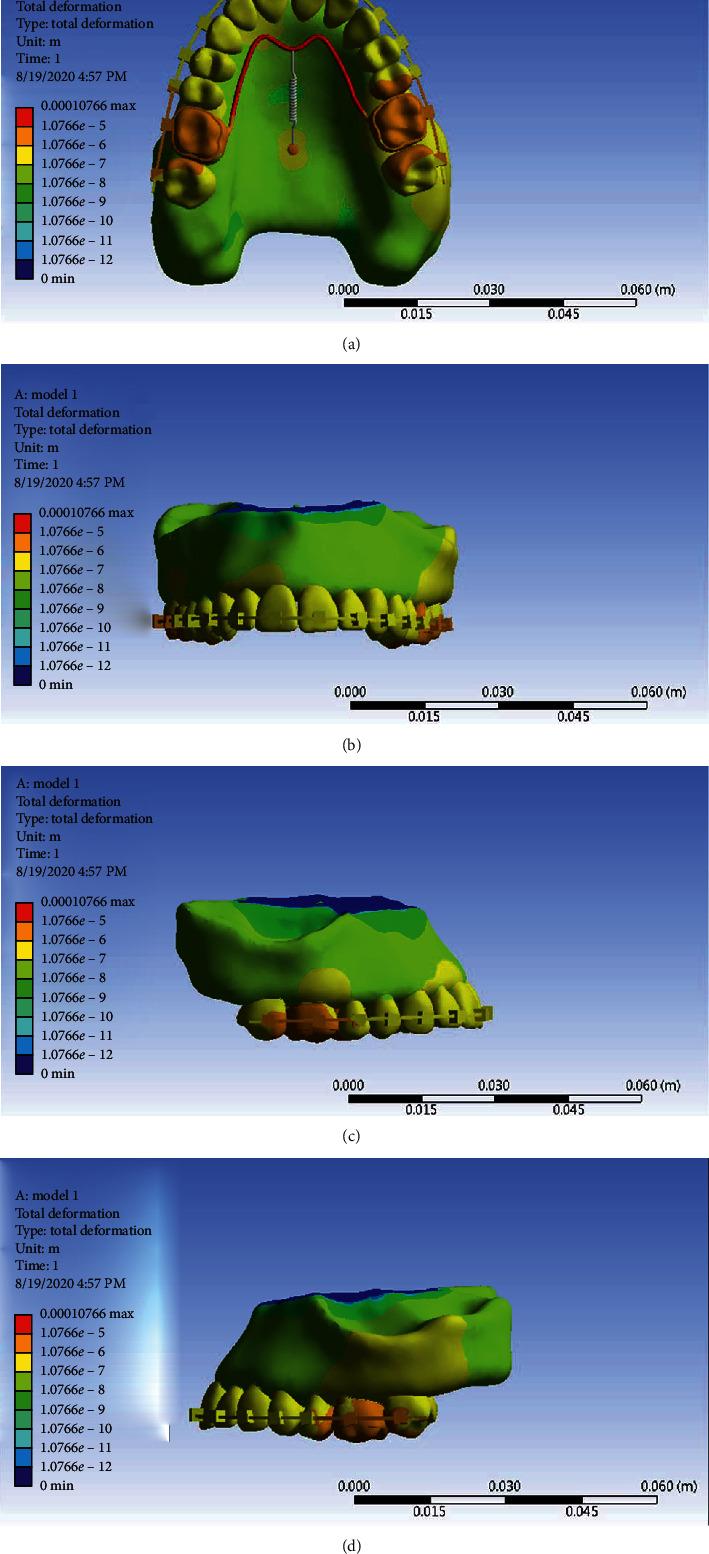
General displacement contour in Model 1 from different angles.

The displacement contour represents the result of movement in all three axes *x*, *y*, *z*. As can be seen from the displacement contour shapes of Model 1 (Figure 19), the most extent of movement is visible in the first molars, mesial sides of second molars, and distal sides of second premolars, which, of course, is quite consistent with previous analyses.

#### 3.3.2. Model 2

**Figure 20:**
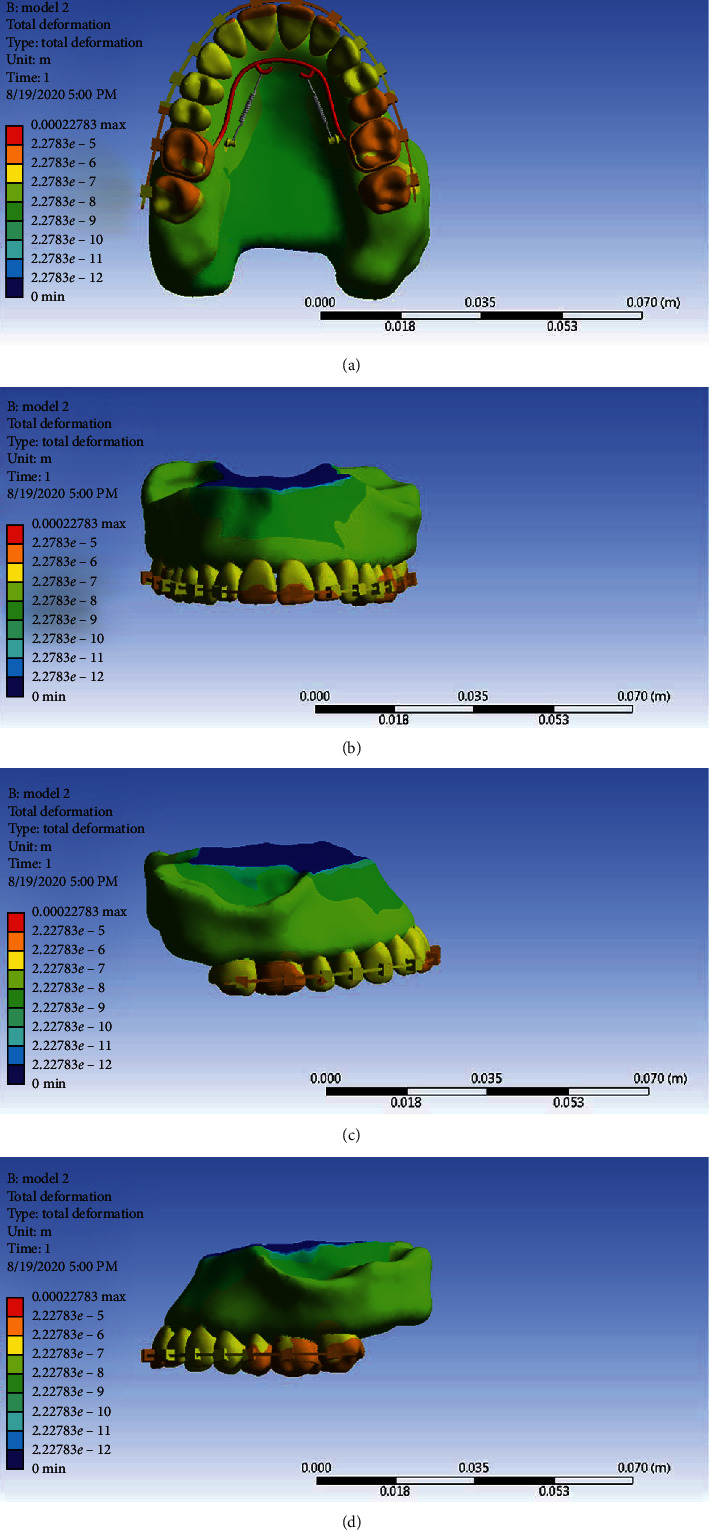
General displacement contour in Model 2 from different angles.

As can be seen from the displacement contours of Model 2 (Figure 20), the amount of displacement in this model is more than Model 1, and the most movement is in the first molars, second premolars and right second molars, mesial sides of left second molars, and finally the incisal edge of incisors.

Displacements of each of the maxillary teeth in 3D in Models 1 and 2 are presented in Tables 2 and 3.

## 4. Discussion

Since there was no study similar to our research, we are bound to discussing more general aspects of this study. En-masse distalization is one of the successful treatment plans to correct the class II relationship [5–7]. Adequate anchorage is an important determinant of treatment outcomes. In this study, the use of a miniscrew implant as a skeletal anchorage was used to distalize the maxillary arch. Miniscrew assisted distalizers are supported with titanium or stainless steel orthodontic miniscrews. These devices present good clinical reliability [34] and excellent mechanical properties also with small diameters [35].

One of the most successful places to place a miniscrew is the buccal and palatal parts of the maxilla. However, the survival rate of implants in the alveolar ridge needs to be improved due to variations in bone quality and the risk of root contact [26]. Interradicular miniscrews need to be repositioned for successful distalization, while the maxillary buccal bone does not have the ideal quality characteristics for miniscrew placement, especially in younger people. But the palatal bone is a safer area with a denser bone for use in mini-implants [27–29]. Midpalatal suture is a very suitable place with suitable keratinized tissue, fully dense bone, and sufficient support to place a miniscrew, while there are no tooth root, nerve, or vessels in this area, while at the same time placing a miniscrew in this place is without the need for surgery. At present, the midpalatal miniscrew can be used with great success for anterior teeth retraction and intrusion, distalization, and protraction of posterior maxillary teeth. Also, this has made it possible to induce dental movements that were impossible or, at best, difficult with conventional orthodontic mechanics [30, 31]. Therefore, we designed two models to perform distalization of the entire upper arch. In one model, we used the conventional interdental miniscrew method in the palatal between the second premolar and first molar, and in another model, a midpalatal miniscrew was used. Comparing the two models, it is evident that in both models, the stress distribution is the highest in the TPA arms and in the head of the miniscrew where the spring is attached. Regarding the displacement in the *X*-axis, the “mesial in” rotation is seen in the first molar of both models. But there is an exception, and that is the “mesial out” rotation of the right second molar. In all measurements, the amount of movement in Model 2 is more than in Model 1. In the *Y-axis*, more tipping is seen in Model 2, especially in the anterior teeth (detorqueing) and the first molar. However, in Model 1, the movement of the first molar is more bodily. Along the *Z*-axis, the intrusion of the mesial side of the first molar and the extrusion of the distal side of this tooth can be seen in both models. Again, the displacement values are higher in the second model. Comparing micromotion and stress distribution of miniscrews in Model 1, maximum stress and micromotion are observed at the head of the miniscrew where it is attached to the spring. Of course, this amount of micromotion increases as time goes by. The same is true for Model 2, but the amount of micromotion is less than what was seen in Model 1. As for the amount of stress, the stress distribution in miniscrews of both models is almost uniform and orange in color, which means that it is seen in approximately 2/3 of the length of the miniscrew. The miniscrew used in the midpalatal suture was thicker and shorter because of the thin soft tissue and more appropriate cortical bone. However, we used longer miniscrews in model 2 because of the thicker soft tissue.

In the stress distribution map during full arch distalization, the maximum stress was in the area of TPA arms and the molar band and neck of the miniscrew. This was in line with the result of the study of Sukjamsri et al. [36] who studied the effect of the location of the miniscrew in the IPANDA distalizing device and also the study of Pratiwi et al. in 2019 [37] on the distribution of stress in the upper first and second molars with TPA in a finite element analysis.

In general, the amount of stress was higher in Model 2, which even reached 534 MP in TPA areas close to the miniscrew. Nevertheless, the maximum stress in the first model was below 100 MP. Of course, the stress distribution in the jawbone in both models was very small and less than the stress threshold for the maxilla jaw, which is 133 MP [38]. Moreover, the amount of stress in the miniscrew of the first model is much more than the second model, and in the neck area of the miniscrew it reached 21.7 MP, while the stress of the miniscrews in the second model was less and below 10 MP. Of course, miniscrews are able to withstand this amount of stress without any damage since the yield stress of titanium is 350 GP (92). All these results are consistent with the study of Ansarat et al. in 2019 [39].

In the study of the amount of miniscrew micromotion in the first model, the most movement was seen in the head and neck area of the miniscrew (2.83*e* − 03 mm to 3.64*e* − 03 mm), and over time, this micromotion increased. But the micromotion of the miniscrews was less in the second model (6.31*e* − 04 mm to 9.44*e* − 04 mm). According to these findings, the midpalatal miniscrew in the first model tends to be easier to withdraw and therefore, the use of this design reduces the risk of breaking the miniscrew and bone destruction at the end of treatment. This finding was also observed in the study of Suzuki et al. [40] in 2011 who sought to examine the amount of torque and stability of miniscrews during installation and removal.

Yamada et al. [41] investigated the effects of distalization of the upper molar using a mini-implant in the buccal region, and their results were consistent with the findings of the present study. However, due to the mechanics of the miniscrew that applied force from the buccal, it caused “mesial out” rotation of the first molar. This result justifies the “mesial in” rotation in the first molar of our study models. This *X*-axis displacement is also reflected in the results of a 2006 study by Kircelli et al. [42] who examined the effects of miniscrew-based distalization using a pendulum. Meanwhile, in our study, there seemed to be a contradiction, and that was the “mesial in” rotation of the second molar in the second model.

In the present study, more tipping was seen in the second model, especially in the anterior teeth, which caused them to detorque, whereas, in the first model, there was more bodily movement, especially in the first molar. This result was also confirmed by the study of Oh et al. [43], who compared distalization of upper molars with and without miniscrew and found that in patients with miniscrew, less tipping and more bodily movement was obtained. Of course, the amount of tipping that was present even in the first model of our study was much less than that in the Oh et al. study [43] in the case of without the miniscrew and more than their result with the miniscrew. It is important to note that the designs of the mechanics were different in the two studies.

In this study, in the distalization of the maxillary arch in both models, we witnessed intrusion in the mesial side of the first molar and extrusion of the distal side of this tooth. Although this happened to the second molar to some extent, it was much less than the first molar. However, in the study by Lai et al. [44], who compared the three methods of a retainer, miniscrew and miniplate in the treatment of dentoalveolar protrusion patients with class 2 malocclusion, introduced the most efficient method as miniplate. Of course, both miniscrew and miniplate methods had intrusion effects during distalization, which was definitely more in the miniplate.

This study was limited by some factors. In silico computer simulations are worthy methods to assess behaviors of tissues under different forces. However, in silico studies do not allow statistical analysis and generalizability. Hence, their results should be verified via in vitro or in vivo designs. Moreover, they cannot simulate all the complex and dynamic forces existing in the oral environment. Therefore, clinical studies should be conducted to validate our results.

## 5. Conclusion

Comparing the two models, it is obvious that in both models, the stress distribution is the highest in the TPA arms and the head of the miniscrew where the spring is connected.

In comparison with the displacement in the *X-axis*, the “mesial in” rotation is seen in the first molar of both models. But there is one exception and that is the “mesial out” rotation of the right second molar. In all measurements, the amount of movement in Model 2 (with palatal interradicular miniscrews) is more than that in Model 1 (with midpalatal miniscrew). In the *Y*-axis, more tipping is seen in Model 2, especially the anterior teeth (detorque) and the first molar, but in Model 1, bodily movement of the first molar is more evident. Along the *Z*-axis, the mesial intrusion of the first molar and the distal extrusion of this tooth can be seen in both models. Again, the displacement values are higher in the second model (with interradicular miniscrews).

In comparison with micromotion and stress distribution of miniscrews, in Model 1, maximum stress and micromotion are observed at the head of the miniscrew where it is attached to the spring. Of course, this amount of micromotion increases over time. The same is true for Model 2, but with a lower micromotion. As for the amount of stress, the stress distribution in both miniscrews of both models is almost uniform and rather severe.

## Figures and Tables

**Table 1:** Material properties assigned to each component.

Component	Young's modulus (GPa)	Poisson's ratio
Dentin	29.9	0.3
Enamel	83	0.3
PDL	6.8 × 10*e* − 4	0.49
Stainless steel	193	0.27
Titanium alloy	113	0.33
Titanium alloy	116.00	0.32
Cortical bone (dense)	14.70	0.30
Trabecular bone (spongy)	0.49	0.30

**Table 2:** Comparison of the displacement of each tooth in Model 1 (in mm) in 3D.

Tooth	*X* plane	*Y* plane	*Z* plane
L7	7.68*e* − 04 mm	2.04*e* − 03 mm	3.33*e* − 03 mm
L6	2.96*e* − 03 mm	1.19*e* − 03 mm	5.14*e* − 03 mm
L5	7.68*e* − 04 mm	1.19*e* − 03 mm	2.43*e* − 03 mm
L4	7.68*e* − 04 mm	1.19*e* − 03 mm	1.53*e* − 03 mm
L3	3.51*e* − 05 mm	3.83*e* − 04 mm	2.78*e* − 04 mm
L2	6.98*e* − 04 mm	3.83*e* − 04 mm	2.78*e* − 04 mm
L1	6.98*e* − 04 mm	3.83*e* − 04 mm	2.78*e* − 04 mm
R1	−6.98*e* − 04 mm	3.83*e* − 04 mm	−2.78*e* − 04 mm
R2	−6.98*e* − 04 mm	3.83*e* − 04 mm	−2.78*e* − 04 mm
R3	−3.51*e* − 05 mm	3.83*e* − 04 mm	−2.78*e* − 04 mm
R4	−3.51*e* − 05 mm	1.19*e* − 03 mm	−2.78*e* − 04 mm
R5	−3.51*e* − 05 mm	2.04*e* − 03 mm	−1.18*e* − 03 mm
R6	−3.63*e* − 03 mm	2.003*e* − 03 mm	−2.99*e* − 03 mm
R7	7.68*e* − 04 mm	2.04*e* − 03 mm	−2.08*e* − 03 mm

L, left; R, right.

**Table 3:** Comparison of the displacement of each tooth in Model 2 (in mm) in 3D.

Tooth	*X* plane	*Y* plane	*Z* plane
L7	1.24*e* − 03 mm	1.19*e* − 03 mm	−4.98*e* − 03 mm
L6	5.44*e* − 03 mm	2.73*e* − 03 mm	9.19*e* − 03 mm
L5	2.64*e* − 03 mm	2.73*e* − 03 mm	3.87*e* − 03 mm
L4	1.24*e* − 03 mm	2.73*e* − 03 mm	2.10*e* − 03 mm
L3	1.24*e* − 03 mm	2.73*e* − 03 mm	3.31*e* − 04 mm
L2	1.49*e* − 04 mm	2.73*e* − 03 mm	3.31*e* − 04 mm
L1	1.49*e* − 04 mm	2.73*e* − 03 mm	3.31*e* − 04 mm
R1	−1.49*e* − 04 mm	3.83*e* − 04 mm	−3.31*e* − 04 mm
R2	−1.49*e* − 04 mm	2.73*e* − 03 mm	−3.31*e* − 04 mm
R3	−1.49*e* − 04 mm	2.73*e* − 03 mm	−3.31*e* − 04 mm
R4	−1.49*e* − 04 mm	3.56*e* − 04 mm	−1.44*e* − 03 mm
R5	−2.94*e* − 03 mm	3.56*e* − 04 mm	−1.44*e* − 03 mm
R6	−7.13*e* − 03 mm	4.28*e* − 03 mm	−4.98*e* − 03 mm
R7	4.04*e* − 03 mm	1.19*e* − 03 mm	−3.21*e* − 03 mm

L: left; R: right.

## Data Availability

All data are available from the authors.
